# Source unreliability decreases but does not cancel the impact of social information on metacognitive evaluations

**DOI:** 10.3389/fpsyg.2015.01385

**Published:** 2015-09-14

**Authors:** Amélie Jacquot, Terry Eskenazi, Edith Sales-Wuillemin, Benoît Montalan, Joëlle Proust, Julie Grèzes, Laurence Conty

**Affiliations:** ^1^Laboratoire de Psychopathologie et Neuropsychologie EA 2027, Université Paris 8, Saint-DenisFrance; ^2^Laboratoire de Neurosciences Cognitives INSERM U960, Ecole Normale Supérieure, ParisFrance; ^3^Laboratoire de Socio-Psychologie et Management du Sport EA 4180, Université de Bourgogne, DijonFrance; ^4^Laboratoire ICONES EA 4699, Université de Normandie, Mont-Saint-AignanFrance; ^5^Institut Jean Nicod, Ecole Normale Supérieure, ParisFrance

**Keywords:** metacognition, social influence, facial expression, confidence, electromyography, epistemic reliability

## Abstract

Through metacognitive evaluations, individuals assess their own cognitive operations with respect to their current goals. We have previously shown that non-verbal social cues spontaneously influence these evaluations, even when the cues are unreliable. Here, we explore whether a belief about the reliability of the source can modulate this form of social impact. Participants performed a two-alternative forced choice task that varied in difficulty. The task was followed by a video of a person who was presented as being either competent or incompetent at performing the task. That person provided random feedback to the participant through facial expressions indicating agreement, disagreement or uncertainty. Participants then provided a metacognitive evaluation by rating their confidence in their answer. Results revealed that participants’ confidence was higher following agreements. Interestingly, this effect was merely reduced but not canceled for the incompetent individual, even though participants were able to perceive the individual’s incompetence. Moreover, perceived agreement induced zygomaticus activity, but only when the feedback was provided for difficult trials by the competent individual. This last result strongly suggests that people implicitly appraise the relevance of social feedback with respect to their current goal. Together, our findings suggest that people always integrate social agreement into their metacognitive evaluations, even when epistemic vigilance mechanisms alert them to the risk of being misinformed.

## Introduction

Other than communicating important information about others’ feelings and attitudes (George and Conty, 2008), non-verbal social cues such as gaze or facial expression also provide circumstantial information that may guide people’s decisions. Remarkably, non-verbal social cues can spontaneously affect metacognitive evaluations of past decisions (Eskenazi et al., in revision). Metacognition refers to the process by which individuals monitor and control their likely success in cognitive tasks (Proust, 2010). As we make a decision, we concurrently monitor our mental activity in order to regulate information processing and behavior (Koriat, 2007). In experimental research, metacognitive evaluations are usually measured by a second-order decision, which may occur in the form of a subjective confidence judgment in past performance on a first-order task (i.e., retrospective evaluations; Fleming et al., 2010; Kepecs and Mainen, 2012). Several works have aimed to identify the informational cues used by people to elaborate their metacognitive evaluations (Alter and Oppenheimer, 2009; Bahrami et al., 2010; Koriat and Ackerman, 2010). In previous work from our lab, we found that people spontaneously adjust their metacognitive retrospective evaluations based on the non-verbal feedback given by another individual (Eskenazi et al., in revision). Here, we investigated whether this form of social influence varies as a function of the reliability of the social source providing the feedback.

In our previous work, we asked people to perform a 2AFC perceptual task and then rate the level of confidence in their responses. Participants’ confidence ratings were higher after another person had oriented his/her gaze toward their response (25% of trials) compared to when the person gazed at the opposite response (25% of trials), or when there was no social cue (50% of trials). Intriguingly, this effect of non-verbal feedback on confidence ratings was present while participants were told that the person’s gaze direction was uninformative and should be ignored (Experiment 1). Furthermore, the effect was observed despite the fact that the person gazed at the participant’s response only half of the time and regardless of response accuracy. Therefore, participants viewed an equal number of trials with objectively correct and objectively incorrect feedback, rendering the person’s gaze direction unreliable for task purposes. Also, using the same experimental design but leading participants to believe that the gaze direction reflected a PP’s response to the same question, the effect was observed at the expense of participants’ metacognitive sensitivity (Experiment 2). Finally, task difficulty (which strongly determines participants’ degree of certainty prior to feedback) did not modulate social influence, which is in contrast to what previous studies may have predicted (Festinger, 1954; Laland, 2004). Therefore, our results suggest that people have a natural tendency to spontaneously assign relevance and trustworthiness to social information, especially when the social information is perceived as offering positive feedback.

In the real world, however, not all social sources are equally reliable. A strong susceptibility of metacognition to social information regardless of source reliability carries a major risk of accidental or intentional misinformation. Developmental studies consistently demonstrate that children trust others selectively starting at 4 years of age. They monitor the informants’ past accuracy and adjust their decisions to the information provided to them (Harris and Corriveau, 2011; Harris et al., 2012; Mills, 2013; Bernard et al., 2015b). We are thus cognitively equipped to evaluate the epistemic reliability of (social) sources of information, a capacity termed ‘epistemic vigilance’ (Sperber et al., 2010). We seem to be able to assign a weight to social information that determines the extent to which we assimilate that information. Here we studied the extent to which beliefs about the epistemic reliability of a social source modulate the influence of non-verbal social information on metacognitive evaluations. Gaging the reliability of an informant mainly consists in gaging the accuracy of the message he/she communicates (Bernard et al., 2015a). The associated mechanisms may rely on a variety of cues, such as the quality of the message, the perceived benevolence of the informant, the number of congruent informants and/or one’s own perceptual or memorial certainty (Sperber et al., 2010; Bernard et al., 2015b). However, to be reliable, the informant must meet a critical condition: he/she must be competent, i.e., possess genuine information (as opposed to misinformation or no information; Fiske et al., 2007; Sperber et al., 2010).

In an amended version of the paradigm described above, participants in the current study performed a first-order 2AFC task, followed by a subjective confidence rating of their own response on each trial. Before reporting their rating, participants saw a short video clip in which an individual either smiled and nodded to express agreement with the participant’s response, frowned and shook his/her head to signal disagreement, or raised his/her eyebrows and shoulders to express uncertainty. The participant was led to believe that these individuals were PPs and that the expression they displayed at each trial reflected whether they had given the same response as the participant (agreement), the opposite response (disagreement), or no response (uncertainty). However, similarly to Eskenazi et al. (in revision), the experiment was controlled so that participants received equal amounts of objectively correct feedback, objectively incorrect feedback, and uncertain feedback. Throughout the experimental session, each participant saw two individuals, one of whom was presented as being more competent at the task than the other. We hypothesized that participants would be more likely to align their confidence with the non-verbal social feedback when it was provided by the more competent individual. We also expected confidence ratings to be higher following positive feedback (agreement) than following disagreement or uncertainty, as our previous results revealed that people were more susceptible to positive/concordant feedback.

In addition to confidence ratings, we collected participants’ facial muscle activity, as an implicit marker of feedback processing. When exposed to facial expressions, people typically display RFRs, which are detectable by EMG and usually match the perceived expression (Bush et al., 1986; Dimberg and Thunberg, 1998; Dimberg et al., 2000; Hess and Blairy, 2001; McIntosh, 2006). The exact mechanisms underlying RFRs remain a hotly debated issue. Several authors have suggested that RFRs reflect the internal simulation of perceived emotions, which facilitates their understanding. In line with this notion, RFRs have been shown to play a role in the elaboration of judgments about perceived facial expression (Niedenthal et al., 2001; Oberman et al., 2007) as well as ratings about one’s emotional reaction to others’ facial expressions (e.g., Sato et al., 2013). Most importantly for the present work, RFRs have been reported to be modulated by the subjective relevance or meaningfulness of the facial expression (Soussignan et al., 2013, 2015). For example, RFRs typically increase for in-group as compared to outgroup members (McHugo et al., 1991; Bourgeois and Hess, 2008; van der Schalk et al., 2011), and when there is potential for interaction with others (Grèzes et al., 2013). Here, we expected to observe greater RFRs to the facial expressions of the competent agent, who by definition provides more reliable feedback than the incompetent agent.

We recorded the EMG activity of participants’ zygomaticus (the facial muscles responsible for pulling the corners of the mouth upward into a smile) and corrugator supercilii (the facial muscles responsible for pulling the brows together). Usually, viewing positive facial expressions elicits increased activity of zygomaticus major muscles, while negative facial expressions evoke increased activity of the corrugator (Dimberg, 1982; Wild et al., 2001; Sonnby–Borgström, 2002; Dewied et al., 2006; Weyers et al., 2006; Sato et al., 2008; Schilbach et al., 2008; Schrammel et al., 2009; Dimberg et al., 2011; Moody and McIntosh, 2011; Rymarczyk et al., 2011). Here, we expected to observe RFRs (i.e., increased zygomaticus activity) in response to facial expressions of agreement (as compared to disagreement and uncertainty), and increased corrugator activity in response to disagreement (as compared to agreement and uncertainty). Furthermore, as markers for the implicit monitoring of competence, we expected RFRs to be enhanced for the competent as compared to the incompetent individual. We also expected these RFR effects to be strongly reflected in the zygomaticus activity, as a reflection of the particular susceptibility to positive social feedback.

## Materials and Methods

### Participants

Twenty-eight volunteers participated in the experiment (14 females; mean age = 24.86 ± 0.79). All reported normal or corrected-to-normal vision and had no neurological or psychiatric history. Each participant gave their written informed consent and received a compensation of 20€. We obtained ethics approval from the local research ethics committee (CPP Ile de France III, approval n° Am5569-1- 2489) for this research. Data from three participants were excluded from the analysis: two failed to correctly identify the competent individual and one reported extreme confidence rating values (see data analysis).

### Stimuli

#### Dots Display Stimuli

The first-order 2AFC task was a number estimation task where participants judged if target displays contained more or fewer dots than a reference display. The displays consisted of arrays of white dots (10-pixel diameter) randomly distributed on a black disk (320-pixel diameter), with at least 10 pixels separating the dots from each other. For target displays, the number of dots varied from 32 to 68 by increments of 4, while the number of dots was fixed at 50 for the reference display. Task difficulty was manipulated by varying the difference in the number of dots separating the target from the reference displays. This difference ranged from ±2 to ±18 dots in five increments, yielding five levels of task difficulty. Using a program in Matlab, we randomly generated 48 different target displays for each distance, as well as 10 different reference displays.

#### Social Stimuli

The social stimuli consisted of 1.5-s videos created for the purpose of the experiment (see Supplementary Methods). Individuals who did not have distinctive features (e.g., mustache, piercing, jewelry, etc.) were filmed wearing black t-shirts against a white background under the same lighting conditions. They were filmed individually and frames contained fontal views that included the top of the head to the shoulders. Several videos were filmed for each expression: agreement (i.e., smiling and nodding), disagreement (i.e., frowning and head shaking), and uncertainty (i.e., raising eyebrows and shrugging). These were non-verbal facial expressions consisting of head movements and were filmed in an ecologically valid context (see Supplementary Methods). A series of pre-tests were conducted to select the videos from two pairs of individuals (one pair of females and one pair of males; mean age = 32.75 years, *SD* = 2.22) who were matched for perceived competence and trustworthiness. We selected three videos from each individual, one for each of the expressions (agreement, disagreement or uncertainty). We paired videos that were judged in pre-tests to be equally persuasive and emotional in the context of our experimental task. Each participant was presented with only one pair of individuals, either the male or the female pair.

### Experimental Procedure

#### Procedure

**FIGURE 1:**
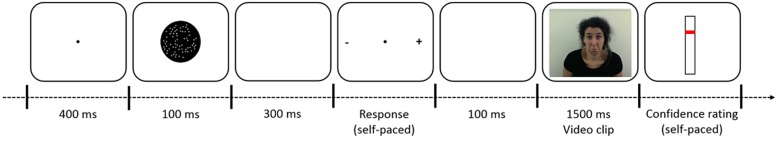
**Time course of an experimental trial**.

Participants were individually tested in a room where they were seated approximately 90 cm away from a 17-inch LCD monitor. Stimulus presentation was conducted using the E-Prime 2.0 software (Psychology Software Tools, Inc., Pittsburgh, PA, USA). Each trial was initiated by a 400-ms presentation of a fixation cross, followed by a brief 100-ms target display. The symbols “-” and “+” appeared on the left and right sides of the screen, respectively, 300 ms after the disappearance of the target, and remained onscreen until the participant gave his/her response. Using a two-choice button, participants had to decide whether the target display contained more (“+”) or fewer (“-”) dots than the reference display. After participants responded, they were presented with a 1.5 s video of a social agent displaying an expression and were asked to indicate their level of confidence in their response using a scale of 0 (not confident at all) to 100 (very confident). The scale remained available on the screen for participants to respond for up to 3000 ms (**Figure 1**). Each participant completed 10 blocks of 24 trials and each block began with a reference display that was presented for 3000 ms. Participants had to keep the reference display in mind to be able to evaluate the upcoming target stimuli.

#### Belief Manipulation

Participants were led to believe that the individuals seen in the videos were actual participants who previously took part in the same experiment. In each trial, the individual’s expression supposedly reflected that “PP’s” answer to the very same dot question. We explained that an expression of agreement would be shown when the PP gave the same response as the participant. If the PP gave the opposite response, the participant would see an expression of disagreement. If the PP did not respond, an expression of uncertainty would appear. To ensure the story’s credibility, participants were filmed before beginning the experiment (wearing a black t-shirt and expressing agreement, disagreement, or uncertainty) and were told that the videos would be used in future sessions of the experiment.

#### Competence Manipulation

At the beginning of the experiment, participants were presented with a picture of the two PPs they would see in the experimental session and each PP’s fictive success rates for the task. These scores were manipulated in order to present one of the PPs as being more competent at the task than the other. They were randomly generated to be between 94.0 and 98.9% for the “competent” PP and between 61.0 and 65.9% for the “incompetent” PP. The two PPs used in each experimental session were always of the same gender and half of the participants were presented with two female PPs while the other half viewed two male PPs. The PP competence associations were counterbalanced across participants.

#### Block Distribution

In order to reinforce the association between PPs and level of competence, the experiment began with two blocks of easy trials (Difficulty 1 and 2). In these induction blocks, the “competent” PP gave correct feedback on 80% of the trials (agreement if the participant gave the correct answer, disagreement if not), incorrect feedback on 10% of the trials (disagreement if the participant gave the correct answer and agreement if not), and expressed uncertainty on the remaining 10% of trials. By contrast, the “incompetent” PP gave correct feedback in only 20% of cases, incorrect feedback in 40% and uncertainty in the other 40%. We included only the easy trials so that participants could easily discern correct from incorrect feedback. During these induction blocks, participants performed 12 trials per difficulty level (2) and PP (2), resulting in 48 trials that were randomly distributed between the two blocks. After the two induction blocks, participants performed three experimental blocks comprising harder trials (difficulty 3, 4, and 5). In these blocks, both PPs provided random feedback, expressing an equal number of agreement, disagreement and uncertainty expressions (i.e., 33%). These three experimental blocks immediately followed the two manipulation blocks so that participants would not notice the change in feedback distribution. The experimental block consisted of 12 trials per level of difficulty (3) and per PP (2). All 72 trials were randomly divided across the three blocks. The entire procedure (two induction blocks and three experimental blocks) was repeated twice. Participants performed 240 trials in total, of which 144 (experimental trials) were analyzed.

#### Post-Test

At the end of the experiment, pictures of the PPs with neutral expressions were presented together on the screen and participants had to choose the most “competent” one. Next, each PP was presented individually and participants were asked to indicate (“yes” or “no”) whether they thought the PP had influenced their confidence, and to what extent (on a scale of 0–3). Participants were also asked to indicate each PPs competence and trustworthiness on a scale of -5 (“not at all”) to 5 (“entirely”).

### Electrophysiological Data Recording and Reduction

We collected surface facial EMG recordings from each participant using the ADInstrument acquisition system (ML870/P Powerlab 8/30). It has been shown that the right hemisphere of the brain is responsible for spontaneous emotional facial reactions (Dimberg and Petterson, 2000), so the EMG electrodes were placed on the left side of each participant’s face.

Throughout the experiment, we continuously recorded *corrugator supercilii* (eyebrow frowning) and *zygomaticus major* (elevation of the mouth corners) muscle activity using Sensormedics 4 mm shielded Ag/AgCl miniature electrodes. Each muscle’s activity was recorded by two electrodes placed on the muscle about 1.25 cm apart (center to center), and roughly parallel to the muscle. The ground electrode was placed at the bottom of the neck dorsally. Before attaching the electrodes, target sites were cleaned with alcohol and rubbed to reduce inter-electrode impedance. The signal was recorded with a sampling frequency of 2 kHz and a band-pass online filter of 500 Hz and then integrated.

Because RFRs were reported to occur during the first second of presentation of a face (Dimberg and Thunberg, 1998; Dimberg et al., 2000; Moody et al., 2007), for each trial of the experimental blocks, we extracted the EMG data collected 300 ms before to 1000 ms after video onset. Integral values were then subsampled oﬄine at 10 Hz, resulting in the extraction of 100-ms time bins. EMG trials containing a noisy baseline (2 SD above or below the mean) were rejected.

Next, the data were log-transformed [Ln (μV)] to reduce the impact of extreme values and standardized (transformed to *Z*-scores) for each participant and for each muscle. Finally, the baseline value (300 ms before video onset) was subtracted from each trial.

### Data Analysis

#### Behavioral Data

Analyses were conducted on the experimental blocks, which included 144 trials in total. Accuracy and reaction times (RTs) for the dot task were submitted to repeated measures ANOVAs using Difficulty (3, 4, 5) as a within-subject factor. A repeated measures ANOVA was conducted on confidence ratings with Competence (Competent vs. Incompetent), Expression (Agreement vs. Disagreement vs. Uncertainty) and Difficulty (3, 4, 5) as within-subject factors. Taking into account the sphericity assumption, we adjusted the degrees of freedom using the Greenhouse–Geisser correction when appropriate (in this case, ε and corrected p values were reported). Planned comparisons were performed when main effects or interactions were observed.

We conducted *t*-tests to compare the two PPs on the different variables recorded during the post-test: Competence, Trustworthiness, and the degree of influence of the PPs. The post-test indicated that two of the 28 participants did not explicitly recognize the competent agent and another individual reported an extreme value for confidence (>2 SD above the mean). All three participants were excluded from the analyses.

#### Electrophysiological Data

Participants with a high rate of trial rejection (2 SD above the mean rate; i.e., >25%) were excluded from the analyses on zygomatic (n = 2) and corrugator (n = 2) activity. The data for each muscle were submitted separately to a repeated measures ANOVA with Competence (Competent vs. Incompetent), Expression (Agreement vs. Disagreement vs. Uncertainty), Difficulty (3, 4, 5) and Time Windows (10) as within-subject factors. Taking into account the sphericity assumption, we adjusted the degrees of freedom using the Greenhouse–Geisser correction when appropriate (in this case, ε and corrected p values were reported). Planned comparisons were performed when main effects or interactions were observed.

## Results

### Behavioral Results

#### First-Order Task

The ANOVAs conducted for performance on the dot task showed a main effect of Difficulty in accuracy [*F*(4,24) = 216.32, ε = 0.73, *p*_corr_ < 0.0001] and in RTs [*F*(4,24) = 36.02, ε = 0.43, *p*_corr_ < 0.0001]. Planned comparisons showed that accuracy decreased (all *p*s < 0.05), while RTs increased (all *p*s < 0.05) with task difficulty (See **Table 1**).

**Table 1:** Accuracy and response time by each level of difficulty for the first order-task (with SD).

	Difficulty 3	Difficulty 4	Difficulty 5
Accuracy (in %)	91 ± 6,6	80.75 ± 8.25	61.25 ± 8.37
Reaction times (in ms)	851 ± 379	1100 ± 416	1374 ± 665

#### Confidence

**FIGURE 2:**
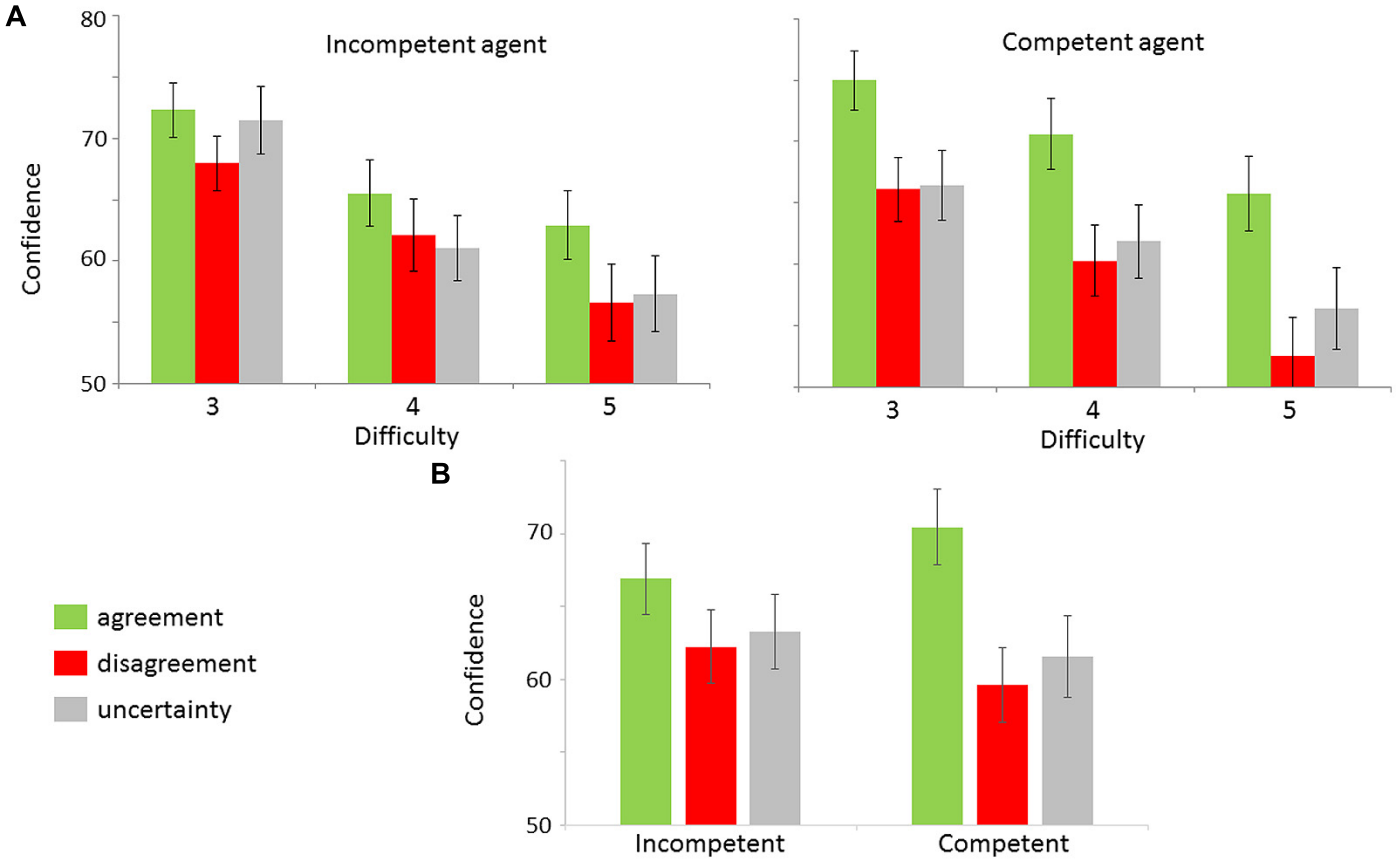
**Confidence levels as a function of experimental conditions. (A)** Mean subjective confidence levels as a function of Competence (Competent vs. Incompetent), Expression (Agreement vs. Disagreement vs. Uncertainty) and Difficulty (3 vs. 4 vs. 5) with SE bars. **(B)** Mean subjective confidence levels as a function of Competence (Competent vs. Incompetent) and Expression (Agreement vs. Disagreement vs. Uncertainty) with SE bars. The data were averaged across different difficulty levels.

The ANOVA indicated a main effect of Difficulty [*F*(2,48) = 38.44, ε = 0.69; *p*_corr_ < 0.0001]. Confidence decreased when task difficulty increased (all *p*s < 0.005). A main effect of Expression was also observed [*F*(2,48) = 22.01, ε = 0.72; *p*_corr_ < 0.0001]. Agreement led to higher confidence than Disagreement [*t*(24) = 5.82; *p* < 0.0001—mean effect size = 7.75 ± 1.51] and Uncertainty [*t*(24) = 5.13; *p* < 0.0001— mean effect size = 6.27 ± 1.30]. Disagreement and Uncertainty did not differ significantly [*t*(24) = 1.85; *p* > 0.05— mean difference = 1.48 ± 0.79], suggesting that Disagreement did not impact confidence in our experimental design. Importantly, we observed an interaction between Competence and Expression [*F*(2,48) = 10.49, ε = 0.91; *p*_corr_ < 0.0001], indicating that agreement expressed by the competent PP has a greater impact on confidence than agreement expressed by the incompetent PP [*t*(24) = 3.78; *p* < 0.001 – **Figure 2**].

#### Post-Test

**FIGURE 3:**
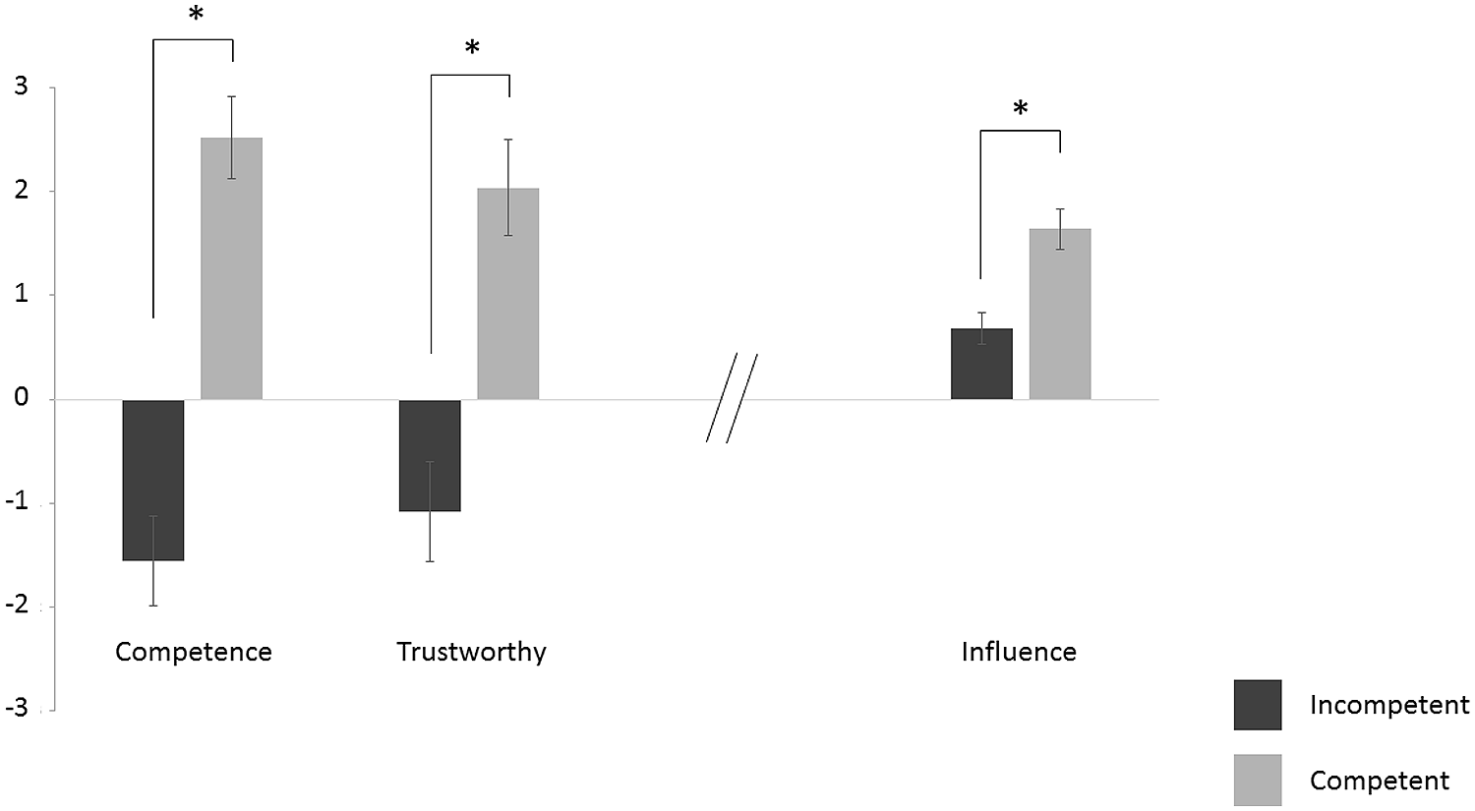
**Post-test results.** Participants’ explicit evaluation of social agents at the end of the experiment with SE bars. The asterisks indicate significant statistical difference.

The *t*-tests revealed that, after performing the task, the competent PP was perceived as being more competent [*t*(24) = 9.05, *p* < 0.0001] but also more trustworthy [*t*(24) = 6.03, *p* < 0.0001] than the incompetent PP (**Figure 3**). Moreover, 88% of participants reported having been influenced by the competent PP, while 52% of participants reported having been influenced by the incompetent PP. The competent PP was also reported to have influenced participants’ confidence more intensely than the incompetent PP [*t*(24) = 4.86, *p* < 0.0001]. A one-sample *t*-test against zero confirmed that participants reported having been influenced by both the competent and the incompetent PPs [all *t*(24) > 4.0, all *p*s < 0.001, **Figure 3**].

### Electrophysiological Results

#### Zygomaticus

**FIGURE 4:**
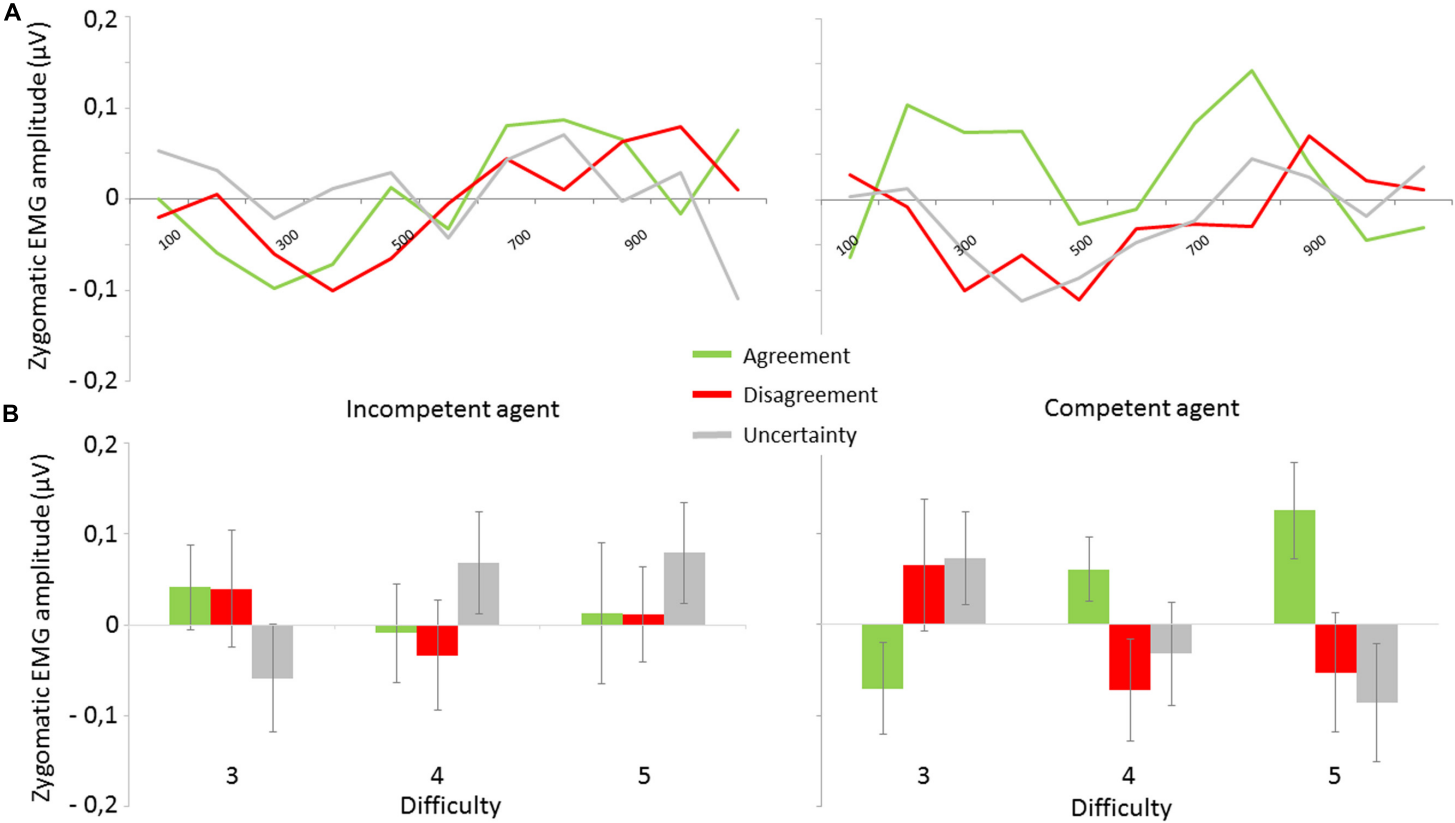
**Zygomaticus activity as a function of experimental conditions. (A)** Time course of mean EMG activity at the onset of the social feedback: mean EMG activity of zygomaticus in μV as a function of Competence (Incompetent vs. Competent) and Expression (Agreement vs. Disagreement vs. Uncertainty). Activity reflects average activation during each 100 ms time interval. **(B)** Mean EMG activity of zygomaticus between 0 and 1000 ms: mean EMG activity of Zygomatic in μV as a function of Competence (Incompetent vs. Competent), Expression (Agreement vs. Disagreement vs. Uncertainty) and Difficulty (3, 4, 5) with SE bars.

The ANOVA did not reveal any main effects of our factors, but a main effect of Time Windows [*F*(9,198) = 2.3, ε = 0.52, *p*_corr_ < 0.05]. However, a three-way interaction among Competence, Expression and Difficulty was observed [*F*(4,88) = 2.6, ε = 0.90, *p*_corr_ < 0.05]. Agreement expressed by the competent PP induced elevated zygomaticus activity when compared to Disagreement expressed by that same PP. This effect was largest for difficulty level 5 [i.e., hardest trials; *t*(22) = 2.16; *p* < 0.05], where Agreement also induced greater activity than Uncertainty [*t*(22) = 2.36; *p* < 0.05]. The difference between Agreement and Disagreement tended to reach significant for difficulty 4 [*t*(22) = 1,74; *p* = 0.09], but disappeared for difficulty 3 [i.e., easiest trials; *t*(22) = 1.44; *p* > 0.1]. Importantly, these modulations were not observed for the expressions of the incompetent PP (all *p*s > 0.2 – **Figure 4**).

#### Corrugators

The ANOVA did not reveal any main effects of our factors, but a main effect of Time Windows [*F*(9,198) = 6.27, ε = 0.46, *p*_corr_ < 0.01]. Moreover, it did not reveal any interactions of our factors on corrugator activity (all *F*s < 2.28; all *p*s > 0.1). We had expected to find corrugator activity in the disagreement condition, as previous experiments have shown an impact of negative emotional displays on this muscle’s activity (Larsen et al., 2003). There are two possible explanations for this lack of corrugator activity modulation: firstly, the lack of impact of disagreement on confidence suggest that this expression was not judged particularly relevant for the task by the participants. Secondly, it is known that corrugators are sensitive to task difficulty (van Boxtel, 2010). Here, the dot task was immediately followed by social feedback, so there may have been a carryover effect of task difficulty on corrugator activity, which would contaminate the effect of feedback. This last possibility limits any further interpretation of corrugator activity patterns.

## Discussion

In this experiment, we investigated whether non-verbal social feedback provided by sources with varying epistemic reliability modulates metacognitive evaluations. To this end, we explored how subjective confidence in performance on a first-order task and RFRs to non-verbal social feedback varied as a function of the reliability of the social source. The results indicated that individuals always integrated social agreement into the elaboration of their metacognitive evaluation, even when mechanisms for epistemic vigilance alert participants to the risk of being misinformed. Albeit to a lesser extent, agreement provided by an unreliable source still impacted participants’ post-decision confidence ratings. However, when asked explicitly, participants were able to distinguish competent informants from incompetent ones. In addition, the RFRs indicated an implicit processing of the competence attributed to the social source.

Regarding subjective confidence, our findings revealed a pattern similar to that reported by Eskenazi et al. (in revision). Participants adjusted their confidence ratings as a function of the information provided by another individual’s non-verbal cues. In Eskenazi et al. (in revision, Experiment 2) participants’ confidence ratings were higher after another person (presented as PP) had oriented his/her gaze toward their response than in the absence of social cues. Here, we found that subjective confidence levels were higher after an individual expressed agreement as compared to disagreement or uncertainty. Eskenazi et al. (in revision, Experiment 2) also found participants’ confidence ratings to be lower after another person had oriented his/her gaze toward the opposite response, compared to when there was no social cue. Here, however, perceived disagreement was not associated with lower levels of confidence than perceived uncertainty. This might be explained by the fact that perceived uncertainty was not neutral; in fact, it may have been sufficient in lowering participants’ confidence. However, we deem it unlikely. That is because any effect of perceived disagreement or uncertainty should have been modulated by source reliability just as the effect of perceived agreement was. The absence of such modulation by source reliability converges toward the view that others’ disagreement and uncertainty were not judged task-relevant by the participants and thus did not impact confidence. This may reveal further that other’s gaze direction is processed in a more reflexive manner than the facial expressions we used in the current study. It is well known that, as soon as 3 months of age, human infants automatically orient their attention toward the direction an adult’s eyes turn (Hood et al., 1998; Farroni et al., 2000; Senju et al., 2006). In adults, such mechanism has been proved to affect automatically our evaluations about object of the environment looked at by others (Bayliss et al., 2006, 2007; Manera et al., 2014). Incongruent gaze direction might be more difficult to ignore than more complex disagreeing facial expressions. Anyway, in Eskenazi et al. (in revision), the effect of congruent gaze was significantly higher than the effect of incongruent gaze. Together with the present results, these findings support the view that positive/concordant information (i.e., agreement) has a stronger effect on confidence judgments than negative/disapproving social information (i.e., disagreement).

Two main (non-exclusive) mechanisms may account for this agreement effect. First, the particular sensitivity to agreement possibly reflects the individuals’ biased tendency to see themselves in a positive light (Leary, 2007) and to expect positive rather than negative feedback (Hepper et al., 2011). It has been suggested that this tendency helps people maintain a positive self-concept (Taylor and Brown, 1988). In other words, the particular susceptibility to social agreement reported here may reflect a self-serving bias. Individuals seemed to reject the validity of the disagreeing feedback, focusing on their potential success while overlooking their potential failures. It is well-known that a self-serving bias heavily influences judgment processes (Fiske et al., 2007). Here, we suggest that a self-serving bias can also influence one’s metacognitive evaluations of past decisions. Alternatively, it is possible that agreements are automatically appraised as being more reliable than disagreement or uncertainty. It is well-known that positive feelings in one area cause other traits to be viewed positively, a form of confirmation bias called the “halo effect” (Thorndike, 1920; Asch, 1946; Nisbett and Wilson, 1977). Consistently, when not manipulated for reliability, all of our videos were judged to be more competent, persuasive, and trustworthy when expressing agreement than disagreement or uncertainty (see Supplementary Methods-Pre-test 3).

Importantly, the impact of agreement or positive social feedback on subjective confidence ratings was greater when it was provided by a competent rather than an incompetent social source. This demonstrates that participants were sensitive to the epistemic reliability of the social source, which modulated the weight they assigned to the social information when elaborating their metacognitive evaluations. Moreover, the post-tests highlighted that the “competent” individual was rated as being more competent as well as more trustworthy. This suggests that competence judgments automatically led participants to calibrate trust as well (Fiske et al., 2007). However, this effect may not be specific to competence. The halo effect predicts that the competent agent was not only judged as more trustworthy, but that he/she was perceived in a more positive light overall than the incompetent agent. This effect may have mediated the greater impact of the competent agent’s agreement on confidence. This implies, for example, that in-group members (who are known to be appraised more positively than out-group members; e.g., Molenberghs, 2013) would have a similar effect on confidence than the competent agent in our study.

Furthermore, electrophysiological results indicated zygomaticus activity in response to agreement when expressed by the competent individual, but only on difficult trials, i.e., when participants were uncertain about their performance on the first-order task. This effect emerged for the medium level of difficulty (Difficulty 4) and reached its maximum for the highest level (Difficulty 5). This suggests that RFRs depend on the reliability attributed to the source, but also on the perceiver’s informational needs. Our physiological results demonstrate first that participants implicitly processed the reliability of the source. They further highlight that they processed social feedback as a function of its relevance to their current goal, such that social cues with high informative value amplified the EMG activity.

RFRs are thought to predominantly reflect the outcome of non-affective motor mimicry (Bavelas et al., 1986; Chartrand and Bargh, 1999), which initially evolved to identify the emotional expression of perceived faces (Hatfield and Rapson, 1993; Niedenthal et al., 2005; McIntosh, 2006) and then to encourage affiliation by favoring liking (Lakin et al., 2003). However, it has also been suggested that RFRs reflect the emotional readout of the perceived facial expression (Cacioppo et al., 1986; Buck, 1994; Dimberg and Thunberg, 1998; Grèzes et al., 2013), which may vary substantially as a function of its relevance to the self (Grèzes et al., 2013; Soussignan et al., 2013, 2015). Our results best fit the second hypothesis. In this study, amplified activity found in the zygomaticus likely reflects the participants’ sense that social agreement indicates a higher probability of success in the task than anticipated (Carver and Scheier, 1990). In other words, it might correspond to the positive experience of having one’s response confirmed by a competent individual – a positive experience that increases with uncertainty about prior performance. The pattern of zygomaticus activity is thus consistent with reports of increased zygomaticus activity with the reward value attributed to smiling faces (Sims et al., 2012) and with arousal level of pleasant facial expressions (Fujimura et al., 2010). The data further suggest that the zygomaticus responses we observed are contingent on the participants’ expectations at each trial.

Interestingly, the social-functional perspective assumes that emotions enable individuals to respond to the situation at hand (Keltner and Haidt, 1999). One may thus expect that the positive experience reflected in the zygomaticus activity contributes to the elaboration of the participant’s confidence judgment, which in turn leads to an increase in confidence. Intriguingly, however, the behavioral data did not follow the same pattern as the RFR results. We observed that positive feedback from both the competent and the incompetent source impacted confidence independently of task difficulty. We might thus speculate that the modulations in confidence we observed reflect an automatic association between another person’s approval and higher subjective confidence in one’s own decision. Another person’s endorsement of one’s own prior response may be motivationally strong enough to raise confidence in that response in a non-analytic manner, even when the source has been presented and appraised as unreliable. We thus speculate that while agreement automatically increases participants’ confidence, the emotional response reflected in the zygomaticus activity depends on context appraisal. This proposal implies that inhibiting RFRs during our experiment would not impact the effect of agreement on confidence. The lack of a clear dissociation between the effects may reveal a discrete role of the emotional reaction (which is reflected in zygomaticus activity) in mediating the impact of social agreement on participants’ confidence.

It is also noteworthy that in the post-test, participants reported having been influenced by the incompetent individual, even though they rated him/her as incompetent and untrustworthy. This is in line with the finding that the implicit processing of social information may be dissociated from explicit beliefs (Chaiken and Trope, 1999; Forgas et al., 2003; Hassin et al., 2005; Bargh, 2006). This further suggests that participants were partly aware of their failure to screen social information as a function of its reliability. They seem to always integrate agreement into the elaboration of their metacognitive evaluation.

Previous studies have shown that individuals have an irrational susceptibility to social feedback, treating it indiscriminately as reliable information (Bahrami et al., 2010; Eskenazi et al., in revision). This may be due to the fact that social feedback is reliable more often than not in natural settings. In line with this notion, others have claimed that cooperation has become an evolutionarily stable strategy that motivates the perception of other participants as knowledgeable and trustworthy partners (Tomasello, 2014). However, although it is generally an adaptive strategy, such social susceptibility can also be detrimental, compromising performance (Bahrami et al., 2010) as well as the accuracy with which performance is evaluated (i.e., metacognitive sensitivity; Eskenazi et al., in revision). The present study advances those findings by demonstrating that individuals are particularly susceptible to positive social feedback, even when they are aware of its unreliability. Here, we propose that this apparently irrational tendency to take on board another’s confirmation when forming metacognitive evaluations is driven by the motivation to maintain a positive self-concept. Moreover, one could further speculate that such self-serving bias has implications for goal achievement. By maintaining a positive self-concept and enhancing confidence, positive social feedback may help individuals engage in the task and devote resources which would eventually improve success (Custers and Aarts, 2005). Likewise, positive reinforcement has been shown to strongly influence learning (Jones et al., 2011).

## Conclusion

Even though we are able to distinguish reliable from unreliable informants both implicitly and explicitly, when elaborating metacognitive evaluations of our past decisions, we are inclined to treat social feedback as reliable when it is confirmatory. Our results further highlight that negative social feedback is not as effective at impacting one’s confidence in oneself. This positively biased processing of social information is robust and may play an instrumental role in social learning that should be addressed in further investigations.

## Conflict of Interest Statement

The authors declare that the research was conducted in the absence of any commercial or financial relationships that could be construed as a potential conflict of interest.

## Abbreviations

2AFC: two alternative forced choice
RFRs: rapid facial reactions
EMG: electromyography
PP: previous participants
SE: standard error
SD: standard deviation
RTs: reaction times.
