# Supplementary material for: Source unreliability decreases but does not cancel the impact of social information on metacognitive evaluations
Source: Front Psychol. 2015 Sep 14;6:1385. doi: 10.3389/fpsyg.2015.01385 (PMC4568399; doi:10.3389/fpsyg.2015.01385)

Supplementary methods

Creation of the feedback stimuli

To make them ecological, the videos were created during an interactive fictive game.

Participants. Eighteen white participants (9 females, ages 25 to 35) born and raised in France, participated in this game. All reported normal vision and had no neurological or psychiatric history. Participants gave their written informed consent to be filmed and have their videos used in an experimental context. At the end of the experiment, participants were debriefed and received a compensation of 20 euros.

Procedure. Upon arrival, participants were first introduced to their so-called “teammates”, who were in fact confederates. Next, the experimenter pretended to place the teammate in an adjacent room. The participants were then invited to wear a black T-shirt and sit down in front of a computer against a white background where lighting conditions were kept the same for all participants. A camera placed above the computer screen filmed participants in a fontal view that included the area from the top of the head to the shoulders.

Participants were led to believe that they were viewing the same computer screen as their teammates and were instructed to help the teammate play a game. The fictive game consisted of identifying the capitals of 40 countries. At every trial, the name of a country appeared on the screen along with the correct and an incorrect but plausible response (example: What is the capital of Morocco? Correct response: Rabat, Incorrect response: Casablanca). After a varying time-lag of 1-4 s, one of the response alternatives was pseudo-randomly highlighted by the program, such that the correct answer was selected in 50% of trials. Participants believed that this response was the one chosen by the teammate, and that at the end of each trial, the camera transmitted their video to the teammate for 3 s. The participants were instructed to use these 3s to indicate to the teammate whether they agreed or disagreed with the response, or whether they did not know the answer. They were also told that the teammate could not hear them but could only see their face. Participants were asked to express their opinion using clear, marked and persuasive facial expressions. To motivate the participants to be persuasive, we told them that they would win 50 Euro-cents each time they succeeded in making the teammate avoid the wrong answer. The participants thus believed that after hearing their response, the teammate would either confirm or change the response, or decide not to answer. Participants were informed that they would not see their teammate’s final decision and that they would find out how much money they won only at the end of the game. In actuality, all participants received a total of 20 Euros.

Video selection: Pre-test 1 on perceived expression and emotional content

We first selected the 10 most expressive individuals among the 18 recorded. We then chose three videos of agreement, three videos of disagreement and three videos of uncertainty from each individual and one other video displaying the individual in a neutral and static position, resulting in a total of 100 1.5 s videos.

Participants. Twenty-four participants (12 females, mean age=25.3±1.4 years) were pre-tested to select the experimental stimuli. All participants reported normal or corrected-to-normal vision

and had no neurological or psychiatric history. At the end of the pre-test, participants received a compensation of 10 Euros.

Procedure. In the pre-test, the videos were presented one by one and followed by two sets of questions. Participants were first instructed to indicate the opinion expressed by the individual by pressing one of the four response buttons (forced-choice procedure): agreement, disagreement, uncertainty, or no opinion. In the case of agreement, disagreement and uncertainty, they also had to rate the intensity of the opinion on a scale of 0 to 9. They then had to indicate the emotion expressed by the individual by pressing one of the three response buttons (forced-choice procedure): positive, negative or neutral. When responding positive or negative, they were also asked to rate the intensity of the emotion on a scale of 0 to 9. The order of presentation of these two sets of questions was randomized across participants.

Analyses. We aimed at selecting stimuli that were of a high quality and would differ as little as possible on the following dimensions: (i) for each video, we first calculated the percentage of correct responses (correct recognition of the facial expression displayed) and then (ii) the mean intensity rating given to each expression by the participants who responded correctly (thus, the number of participants varied slightly between videos on this last variable). For each video, we also calculated (iii) the valence attributed to the expression as a percentage (negative, neutral or positive) and (iv) the mean intensity of the video's emotional content as judged by the participant for the two more frequent responses (thus, the number of participants varied slightly between videos on this last variable). We then selected one video of each expression (agreement, disagreement or uncertainty) from two pairs of individuals from each gender based on the quality of the expressions and their emotional content (see table S1). Finally, we conducted t-tests to compare the selected videos on each variable, separately for each expression and each pair of individuals.

Results. As defined above, agreement videos show the individuals smiling and nodding yes, disagreement videos show the individuals frowning and shaking their head, and uncertainty videos show the individuals raising their eyebrows and shoulders. Accordingly, agreements were predominantly judged to be emotionally positive, disagreements emotionally negative, and uncertainty videos were deemed neutral (see Table S1 for more details). However, qualitatively, the agreement of Woman 1 was less often categorized as having positive emotional content than the agreement of Woman 2. The uncertainty of Woman 1 was more often categorized as having positive emotional content than the uncertainty of Woman 2. For the male pair, the disagreement of Man 1 was less often categorized as having negative emotional content than the disagreement of Man 2 (See Table S1). For a given expression and a given pair, the videos were rated with similar emotional intensity (Table S1), except for the uncertainty expressed by the female individuals and the disagreement expressed by the male individuals. The uncertainty of Woman 1 was judged more positive than the uncertainty of Woman 2 ($t(41)=2.46$; $p<.05$, Table S1). The disagreement of Man 2 was judged more negative than the disagreement of Man 1 ($t(43)=3.41$; $p<.005$, Table S1). Importantly, for a given pair, the agreement and disagreement videos were rated with similar emotional intensity (all $ps>.1$), while the videos of uncertainty were rated less emotionally intense (all $ps<.05$). Also importantly, all the expressions were correctly categorized and rated with similar intensity (see Table S1).

Pre-test 2 on competency and trustworthiness

Participants. A total of 22 participants (11 females, mean age = 20.9±.3 years) took part in the experiment. All reported normal vision and had no neurological or psychiatric history. Participants gave their written informed consent.

Procedure and results. In the second pre-test, we presented the neutral videos of the four selected individuals, one by one. Participants were then asked to indicate how competent and trustworthy they deemed the individuals on a scale of -5 to 5. Competence (females: t(21)=0.11, p>.05, males: t(21)=1.82, p>.05) and trustworthiness (females: t(21)=1.44, p>.05; males: t(21)=1.3, p>.05) judgments did not differ between pairs (see Table S1).

Supplementary Table S1. Mean scores with standard errors obtained by each PP and each video on the first 2 pre-tests.

* Regarding the valence of the video's emotional content, participants had a 3 forced choice task (positive-negative-neutral). Below the percentage of the two majority responses is presented.

** The mean intensity of the video's emotional content has been computed from the two majority responses. One of them was always the neutral option that was automatically associated with an intensity of 0. Intensities related to the third response option have been rejected from the analyses.

	Woman 1				Woman 2			
Competent	1.72±0.37				1.68±0.34			
Trustworthy	1.59±0.40				2.10±0.29			
			*	**			*	**
	% Correct Recognition of Expression	Intensity of the Expression	Valence of the emotion	Intensity of the emotion	% Correct Recognition of Expression	Intensity of the Expression	Valence of the emotion	Intensity of the emotion
Agreement	95.83%	6.79 ±0.32	Positive: 66.67% Neutral: 29.17%	2.83 ±0.53	95.83%	6.63 ±0.52	Positive: 83.33% Neutral: 16.67%	3.67 ±0.56
Disagreement	95.83%	7.29 ±0.34	Negative: 83.33% Neutral: 8.33%	4.77 ±0.53	100%	7.50 ±0.40	Negative: 75% Neutral: 20.83%	3.48 ±0.55
Uncertainty	95.83%	7.29 ±0.29	Positive: 54.17% Neutral: 41.67%	2.82 ±0.6	95.83%	7.75 ±0.50	Neutral: 62.5% Positive: 20.83%	0.85 ±0.35

	Man 1				Man 2			
Competent	1.54±0.32				1.95±0.29			
trustworthy	1.63±0.36				1.05±0.4			
			*	**			*	**
	% Correct Recognition of Expression	Intensity of the Expression	Valence of the emotion	Intensity of the emotion	% Correct Recognition of expression	Intensity of the expression	Valence of the emotion	Intensity of the emotion
Agreement	91.67%	6.21 ±0.39	Positive: 83.33% Neutral: 16.67%	3.75 ±0.49	100%	6.00 ±0.51	Positive: 83.33% Neutral: 8.33%	4.45 ±0.48
Disagreement	91.67%	6.38 ±0.45	Negative: 50% Neutral: 37.5%	2.52 ±2.61	100%	7.13 ±0.42	Negative: 91.67% Neutral: 8.33%	5.13 ±0.5
Uncertainty	91.67%	6.00 ±0.54	Neutral: 58.33% Positive: 16.67%	1.4 ±0.51	91.67%	7.04 ±0.51	Neutral: 79.17% Positive: 12.5%	0.68 ±0.42

Pre-test 3 on persuasiveness, emotions, competency and trustworthiness, in the context of the experimental task

Since the first pre-test revealed some asymmetries in the videos' emotional content, we ran a last test in which the participants judged the videos on several dimensions of interest, but in the context of the experimental task. The purpose was to determine whether for a given expression, context and pair of individuals, the videos were perceived as equivalent in terms of competency and trustworthiness of the PP, persuasiveness of the videos and triggered emotions.

Participants. Twenty-two participants participated in this behavioral experiment (11 females; mean age = 22.36 ± 4.02). All reported normal or corrected-to-normal vision and had no neurological or psychiatric history. Each participant gave his/her written informed consent and received a compensation of 5 euros.

Procedure. The procedure was the same as that of the main experiment with one difference: here, participants saw two trials (48 dots and 52 dots, i.e, the highest level of difficulty: difficulty 5) for each of the three expressions (Agreement, Disagreement, and Uncertainty) expressed by four PPs (2 females and 2 males). In total, they evaluated 24 videos distributed across two blocks. We included only the hard trials so that the participants could not discern correct from incorrect feedback. The purpose was to encourage the participants to base their judgments on the videos' features rather than on their objective reliability.

As in the main experimental procedure, each trial was initiated by a fixation cross that was presented for 400 ms, followed by a brief target display presented for 100 ms. 300 ms after the disappearance of the target, the symbols "-" and "+" appeared on the left and right sides of the screen, respectively, and remained until the subject responded. Participants used a two choice button to indicate whether the target display contained more ("+") or fewer ("-") dots than the reference display. After responding, participants were presented with a 1.5 s video of a social agent displaying an expression and were asked to respond to four questions presented in random order. They were asked to indicate how competent and trustworthy they found the PP using a scale of -5 ("not at all") to 5 ("entirely"). Next, they were asked to rate the level of persuasiveness of the video on a scale of -5 ("not at all") to 5 ("entirely"), and what emotion it provoked in them, again on a scale of -5 ("negative emotion") to 5 ("positive emotion").

Data Analysis. A repeated measures ANOVA was performed on participants' responses with Expression (Agreement vs. Disagreement vs. Uncertainty) and PP (4 different actors) as within-subject factors. Taking into account the sphericity assumption, we adjusted the degrees of freedom using the Greenhouse-Geisser correction when appropriate (in this case, ε and corrected p values were reported). Planned comparisons were performed when main effects or interactions were observed. We also reported accuracy on the 2AFC task.

Results. Participants performed the dot task with an accuracy of 51% ± 0.08. As expected, they were at chance level during the pre-test, suggesting that they were not able to discern correct from incorrect feedback. The ANOVA for video evaluations indicated a main effect of Expression regarding the competence of the PP ($F(2, 42)=32,1$, $\varepsilon = 0.89$; $p_{corr}<.0001$), the trustworthiness of the PP ($F(2,42)=19,01$, $\varepsilon = 0.85$; $p_{corr}<.0001$), and the persuasiveness of the video ($F(2, 42)=28,63$, $\varepsilon = 0.95$; $p_{corr}<.0001$). These main effects revealed that individuals were judged as being more competent (all ps < .0001) and trustworthy (all ps < .0001) when they expressed agreement than when they expressed disagreement or uncertainty (all ps < .0001). The videos portraying agreement were also judged to be more persuasive (all ps < .001). Importantly, all of these main effects were not modulated by the PPs. For a given expression, all PPs were rated equally on all the dimensions of interest (Supplementary figure 1). Interestingly, the ANOVA also revealed a main effect of Expression on the emotional feelings triggered by the videos ($F(2,42)= 42,2$, $\varepsilon = 0.88$; $p_{corr}<.0001$). The videos portraying agreement were reported to trigger stronger positive feelings relative to the other videos (ps < .001 for all PPs). The videos portraying disagreement triggered more negative feelings relative to uncertainty ($t(21)=2,52$; $p<.05$). Importantly, for a given expression, all PPs were rated equally on emotional intensity, except the disagreements of the couple of men (interaction between the Expression and the PPs: $F(6, 126)=2,57$, $\varepsilon = 0.62$; $p_{corr}<.05$). The video of Man 1 was rated less negative than the video of Man 2 $t(21)=2,76$; $p<.02$ – see Figure S1).

Discussion. Although pre-test 1 revealed some asymmetries in the emotional content of the selected videos, the present results emphasise that for any given expression, all videos were rated equally on all the dimensions of interest (except the videos of disagreement of Man 1 and 2 that differ in negative intensity). The results of pre-test 3 also revealed a strong impact of agreement on videos and individuals' processing. They converged toward the view that we are positively biased towards those who favour us (Jones et al., 2011). They also emphasised that the perception of an agreement triggers positive feelings, the perception of a disagreement triggers negative feeling, while an uncertainty was associated with a neutral emotional state.

Supplementary Figure S1. Mean of ratings with standard error bars obtained by each PP and each video at each dimension of the third pre-test. W=Woman, M=Man.

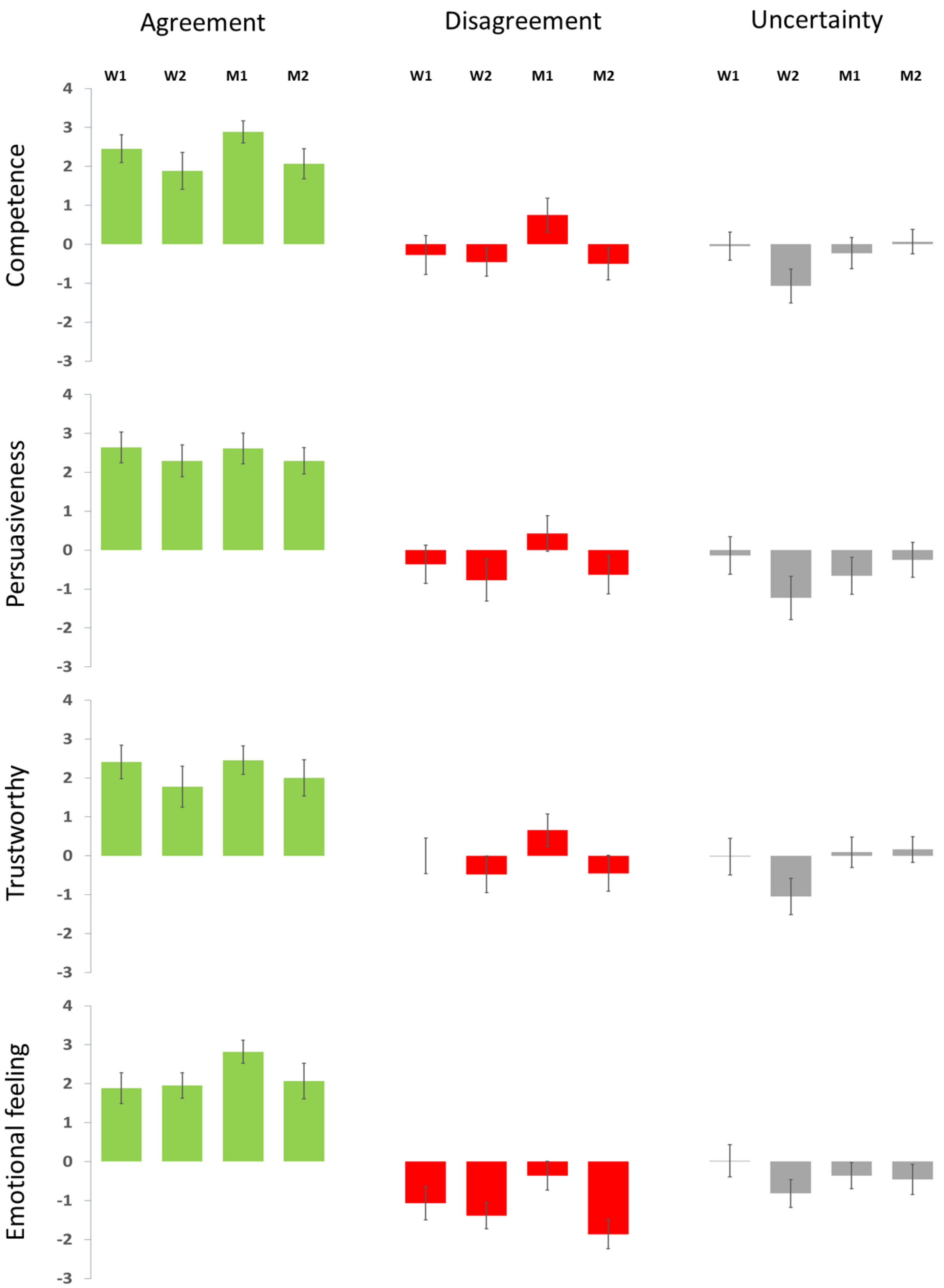

Supplement: Supplementary file 1 [file Data_Sheet_1.PDF]
